# Chromoendoscopy in colorectal surveillance for primary sclerosing cholangitis and inflammatory bowel disease

**DOI:** 10.1055/a-2518-9380

**Published:** 2025-02-05

**Authors:** Rodrigo V Motta, James E East, Emma L Culver

**Affiliations:** 1 6396 Translational Gastroenterology and Liver Unit, Experimental Medicine Division, Nuffield Department of Medicine, University of Oxford, Oxford, United Kingdom of Great Britain and Northern Ireland



We would like to thank Nishad et al. for their insightful comments regarding our recently published manuscript 1 demonstrating the superiority of dye-based chromoendoscopy (CE) compared with white light endoscopy for neoplasia detection in patients with primary sclerosing cholangitis-inflammatory and bowel disease (PSC-IBD) and highlighting the important role of CE to detect subtle serrated lesions. Patients with PSC- IBD are a select group with much higher risk of colorectal cancer (CRC) than the IBD-only population 2 .

Of the 359 colonoscopies performed in our cohort, 61% were performed by a consultant with extensive experience in CRC screening, 24% by a senior clinical fellow with more than 5 years of experience, and the remainder were done by a combination of nurse endoscopists and senior specialty registrars with supervising consultants. Random biopsies were obtained in 340 procedures (94%), there were 27 post-inflammatory polyps, and 59 neoplastic lesions were identified among those extracted using endoscopic mucosal resection. We did not have reliable data on family history of CRC to perform further analysis as suggested, such as inverse probability of treatment weighting.

It is important to focus on optimal techniques for neoplasia detection, given the increased incidence of non-conventional lesions 3 , and indeed, artificial intelligence will play a role here. We welcome a recent network meta-analysis that highlights the benefit of chromoendoscopy in IBD even in the era of high-definition white light, which correlates with the benefit we observed in PSC-IBD 4 .

**Publication note**

Letters to the editor do not necessarily represent the opinion of the editor or publisher. The editor and publisher reserve the right to not publish letters to the editor, or to publish them abbreviated or in extracts.
